# Gene Expression Analysis in Patients with Cocaine-Induced Midline Destructive Lesions

**DOI:** 10.3390/medicina57090861

**Published:** 2021-08-24

**Authors:** Matteo Trimarchi, Giacomo Bertazzoni, Alessandro Vinciguerra, Celia Pardini, Fabio Simeoni, Davide Cittaro, Mario Bussi, Dejan Lazarevic

**Affiliations:** 1 Department of Otorhinolaryngology, IRCCS San Raffaele Scientific Institute, Vita-Salute San Raffaele University, 20132 Milan, Italy; vinciguerra.alessandro@hsr.it (A.V.); bussi.mario@hsr.it (M.B.); 2 Department of Otorhinolaryngology, Azienda Socio-Sanitaria Territoriale di Cremona, 26100 Cremona, Italy; giacomo.bertazzoni@asst-cremona.it; 3 Center for Omics Sciences, IRCCS San Raffaele Scientific Institute, 20132 Milan, Italy; pardini.celia@hsr.it (C.P.); simeoni.fabio@hsr.it (F.S.); cittaro.davide@hsr.it (D.C.); lazarevic.dejan@hsr.it (D.L.)

**Keywords:** paranasal sinus disease, craniofacial region, chronic disease, cocaine, CIMDL

## Abstract

Background and Objectives: Cocaine users may present with positive antineutrophil cytoplasmic antibodies (ANCA) and severe midline destructive lesions (CIMDL) which are histologically characterized by massive apoptosis. However, histopathological and laboratory studies suggest that autoimmunity may not be the main pathogenic driver. We analyzed gene expression both in cell lines of nasal mucosa exposed to cocaine and in CIMDL patients to determine whether genetic predisposition might cause such lesions, which are observed in a minority of cocaine abusers. Materials and Methods: The genetic expression profile of nasal mucosa exposed to cocaine was analyzed. Rare variants of expressed genes were searched in patients with CIMDL using exome sequencing and bio-informatics. Results: We identified 462 genes that were induced by cocaine, mainly related to apoptosis and autophagy in response to oxidative stress. Under the hypothesis that genes linked to the phenotype are also induced by cocaine itself, a rare variants burden test was performed to select genes that were significantly enriched in rare mutations. Next, 11 cocaine abusers with CIMDL and no other relevant medical comorbidities underwent exome sequencing, and 12 genes that were significantly enriched in the burden test and present in at least 10 patients were identified. An in-depth analysis of these genes revealed their involvement in apoptosis, tissue homeostasis, autophagy, and response to oxidative stress. Conclusions: Oxidative stress and rare genetic alterations in the response to reactive oxygen species, apoptosis, autophagy, and tissue regeneration are plausible drivers of damage affecting nasal mucosa exposed to cocaine crystals and, consequently, the pathogenic mechanism behind CIMDL.

## 1. Introduction

**Figure 1:**
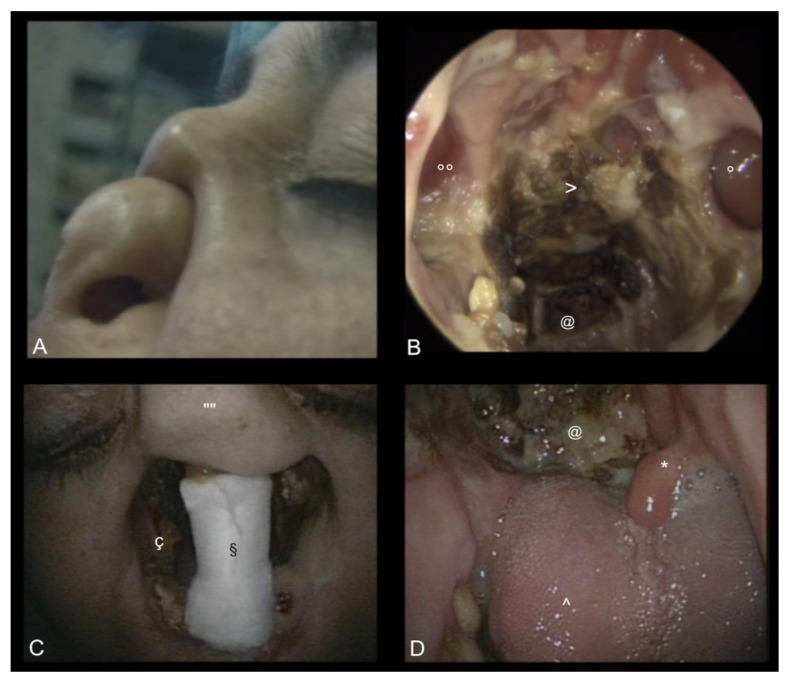
The picture shows the extent of cocaine-induced damage to the nasal dorsum (**A**), columella (**C**, §), and superior lip (**C**; ç indicates erosion of the piriform aperture; “” marks the remaining nasal dorsum). Pictures (**B**,**D**) show the endoscopic endonasal view of the oro-nasal cavity of the case illustrated in picture (**C**), which shows the total erosion of nasal septum, inferior, middle, and superior conchas, hard palate, sub-total erosion of soft palate (* shows the remaining uvula), exposure of the skull base (@ and >), as well as opening of the maxillary sinuses (° and °°).

Cocaine is the most commonly used illicit stimulant drug in Europe [1]. Estimates of the European Monitoring Centre for Drugs and Drug Addiction (EMCDDA) indicate that about 2.6 million young adults (aged 15 to 34) used cocaine during the previous year [1]. Intranasal cocaine abuse is known to cause damage to nasal mucosa, along with potentially fatal systemic effects and addiction [2,3]. In some habitual cocaine users, however, damage to nasal structures can extend to the underlying osseous and cartilaginous structures of the nose, resulting in cocaine-induced midline destructive lesions (CIMDL) (Figure 1) [2,4]. While the exact prevalence of CIMDL is unknown, a search of the US National Library of Medicine (PubMed) online database from 1 January 1982 to 31 December 2019 found 114 reports describing roughly 200 patients; however, this number is in stark contrast with data from the US Substance Abuse and Mental Health Services Administration, which estimated that in 2018 about 5.5 million US residents used cocaine [5]. In fact, if CIMDL were simply the last stage of chronic cocaine-induced mucosal damage and erosion, it would be expected that these lesions would be much more prevalent among habitual users, even if the possible role of cutting substances is taken into account. Moreover, our clinical experience suggests that individuals exposed to the same drug batches at similar doses do not necessarily develop lesions with the same frequency or severity [1]. 

Since the clinical appearance of CIMDL overlaps with upper respiratory tract granulomatosis with polyangiitis (GPA), autoimmunity has been suggested as a possible cause [2]. However, histopathological and laboratory studies conducted to date suggest that cocaine-induced apoptosis and not autoimmunity may be the main driver of those lesions [2]. Moreover, cocaine is known to induce the expression of genes with a role in response to oxidative stress and DNA damage [6,7]. Based on these findings, to further elucidate the pathogenesis of CIMDL we analyzed the genetic expression of nasal mucosa samples exposed to cocaine. Expressed genes were then examined in patients with CIMDL using whole exome sequencing and bioinformatics analysis to identify the main biomolecular mechanisms involved in CIMDL and confirm the expression of genes related to apoptosis and oxidative stress response. 

Secondly, considering the discrepancy between the number of cocaine abusers and the relative low frequency of CIMDL we hypothesized that genetic predisposition could play a role in CIMDL expression. Therefore, we tried to find rare variants of the identified cocaine-induced genes that could be linked to the destructive effect of cocaine abuse. We then attempted to verify the expression of the identified rare variants in a sample of CIMDL patients. 

## 2. Materials and Methods

### 2.1. Cell Culture

The nasal epithelial immortalized cell line RPMI 2650 from ATCC^®^ was used as an in vitro nasal model.

### 2.2. Reagents

Cocaine base (LGC, Teddington, UK) was prepared as 50 mM solution (50% Phosphate Buffered Saline (PBS)/50% ethanol). Staurosporine, a known inducer of apoptosis, was employed as a positive control, prepared as 15 mM in Dimethyl Sulfoxide (DMSO).

### 2.3. Experimental Design

In order to determine the optimal time window and treatment conditions, we performed a preliminary experiment using two different treatment times (1 and 6 h), different concentrations of cocaine (0.1 µM, 1 µM, 2 µM, 3 µM, 4 µM, 5 µM) and staurosporine (1 nM, 5 nM, 10 nM), along with two negative controls (cell culture medium alone, and cell culture medium with 50% of PBS/ethanol, 1:1). After the incubation period, cells were washed and incubated without drug for 24 or 48 h, respectively. Starting from 3 µM cocaine, massive apoptosis was observed in both short and long treatment periods (data not shown).

The experiment was then repeated under the following conditions: incubation for 6 h at 37 °C in the presence of either cocaine (0.1 µM, 1 µM, 2 µM, 3 µM, 4 µM, 5 µM) or staurosporine (1 nM, 5 nM, 10 nM), and two negative controls (see above), followed by washing, cell harvesting, and RNA extraction after 24 h. All cells were plated in duplicate (technical replicates) for each time point to determine toxicity and to extract RNA. All experiments were done in triplicate.

### 2.4. RNA Extraction and High-Throughput Sequencing (HTS)

RNA from treated cells and control samples was extracted using the RNeasy Mini Kit ^®^ (Qiagen, Hilden, Germany). Quality and quantity of total RNA were evaluated by running samples onto Tapestation 4100^®^ (Agilent, Santa Clara, CA, USA) and Qubit^®^ (Thermofisher, Waltham, MA, USA), respectively. Libraries were prepared for HTS sequencing using the TruSeq^®^ mRNA stranded kit (Illumina, San Diego, CA, USA) following the manufacturer’s protocol starting from 100 ng of total RNA. Sequencing was performed using Novaseq 6000^®^ (Illumina, San Diego, CA, USA) in SE mode, generating in average 30 M reads per sample, 100 nt long. 

### 2.5. RNA-Seq

Read tags were pseudo-aligned to GENCODE transcripts v27 [8] using Kallisto v0.44.0 (Pachter Lab, Pasadena, CA, USA). [9] Transcripts were summarized to genes using the tximport [10] package (Bioconductor, Buffalo, NY, USA). Differential expression was evaluated using limma (Bioconductor, Buffalo, NY, USA) [11] interpolating the dose of chemical (cocaine or staurosporine) with a spline curve using two degrees of freedom. Genes induced by medium (PBS + ethanol) were estimated by a comparison with untreated cells. *p*-values were corrected using qvalue package (Bioconductor, Buffalo, NY, USA) [12]. Genes were considered significant at q < 1 × 10^−3^. Genes that were found to be regulated by cocaine but not by staurosporine or PBS-ethanol were considered for investigation.

### 2.6. Exome Sequencing

Exome sequencing was performed on blood samples drawn from CIMDL patients who had been referred to the Otorhinolaryngology Department of San Raffaele Hospital (Milan, Italy). All patients gave informed consent and the study was conducted according to the principles of the revised Declaration of Helsinki, in compliance with Good Clinical Practice and ethical standards, and was approved by local ethics committee (Comitato Etico dell’Ospedale San Raffaele). 

Read tags were aligned to reference genome hg19 using BWA MEM (Illumina, San Diego, CA, USA) [13] and duplicated reads were identified using Samblaster (GitHub, San Francisco, CA, USA) [14]. Variant calling was performed using Freebayes [15] Variant Call Format (GitHub, San Francisco, CA, USA) was annotated for functional impact using snpEff (Paolo Cingolani, Arlington, MA, USA) [16]. Variants were filtered for quality (QUAL > 1, RPL > 1, RPR > 1, SAF > 0, SAR > 0, MQM > 50, MQMR > 50) and for impact on the protein sequence (MODERATE or HIGH impact). Variants were further filtered for allele frequency lower than 1% in dbSNP v146 (National Center for Biotechnology Information, Bethesda, MD, USA) (coded allele frequency < 0.01). Burden test was performed using TRAPD (GitHub, San Francisco, CA, USA) [17] using the Exome Aggregation Consortium data as controls. *p*-values were corrected by False Discovery Rate [18].

## 3. Results

**Figure 2:**
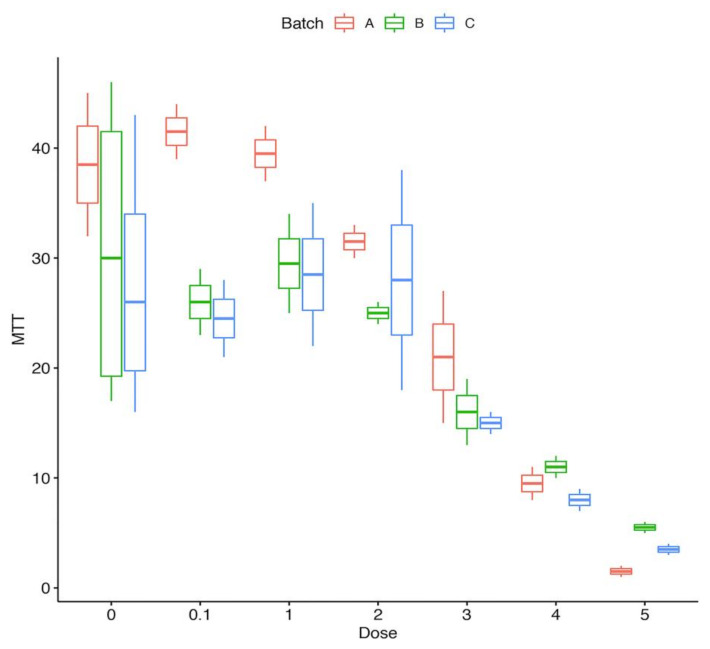
Effect of cocaine on cell growth. The boxplot shows the cell vitality, measured by 3-(4,5-dimethylthiazol-2-yl)-2,5-diphenyltetrazolium bromide (MTT), at increasing dosage of cocaine. We present data for three experiments, each in triplicate (A, B, and C).

We performed RNA sequencing to identify genes that were regulated by incubation of cocaine with cultured cells. To do so, we tested increasing concentrations of cocaine (0.1 µM, 1 µM, 2 µM, 3 µM, 4 µM, 5 µM); we also tested increasing concentrations of staurosporine (1 nM, 5 nM, 10 nM) as control for apoptosis. Indeed, increasing the concentration of cocaine inhibited cell growth (*p* = 1.29 × 10^−7^), occurring at cocaine concentrations as low as 3 µM (Figure 2). 

**Figure 3:**
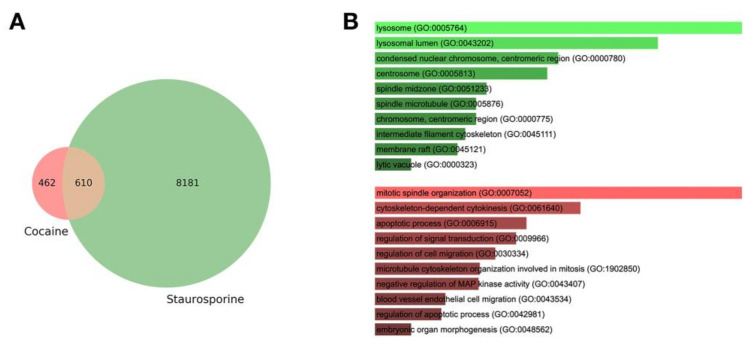
(**A**) Differentially expressed genes upon cocaine administration. The Venn diagram shows the number of genes that are regulated at increasing dose of cocaine or staurosporine. Genes were selected at q < 1 × 10^−3^; out of 1072 genes, only 462 were found specific for cocaine. (**B**) Gene Ontology analysis. The bar chart shows the top 10 GO:BP (gene ontology:biological process) categories enriched for the genes specifically induced by cocaine. A large fraction of terms is related to apoptosis.

We identified 462 genes that were induced by cocaine in a specific manner (Figure 3A). Analysis of Gene Ontology terms showed an enrichment in processes and compartments mainly related to apoptosis and autophagy/lysosomal activity (Figure 3B). Under the hypothesis that genes linked to the clinical phenotype are also induced by cocaine, a rare variants burden test was performed to select genes that were significantly enriched in rare mutations compared to a control population. 

Following this, in order to identify genes possibly associated with CIMDL we performed exome sequencing on a set of 11 cocaine abusers with CIMDL (six females and five males). Age and degree of facial destruction caused by CIMDL are reported in Table 1. 

All patients sought medical care for CIMDL-related symptoms and had no other relevant medical comorbidities. Twelve genes that were significantly enriched in the burden test and present in at least 10 patients were identified: AHNAK, C1orf116, CACHD1, FBN1, IQGAP2, OSGIN1, PARP4, PDLIM5, PPP1R15A, PVR, TBC1D2, and ZNF469. Interestingly, all genes were found to be induced and none repressed by cocaine, suggesting a possible mechanism of loss-of-function. An in-depth analysis of these genes revealed their involvement in apoptosis, tissue homeostasis, autophagy, and response to oxidative stress (Table 2).

## 4. Discussion

Intranasal cocaine is a well-known irritating factor for the nasal mucosa, but in only a small percentage of habitual abusers the damage extends to the underlying structures causing CIMDL. Several studies have been conducted trying to understand the predisposing factors of these type of lesions, with no common consensus achieved [2,3]. To date, several aspects make research on CIMDL complex, namely the unreliability of abusers’ reporting, the difficulty in determining the exact substance concentration, length of use, frequency, and quantity administered [4,7]. Nonetheless, our clinical experience and evaluation of current epidemiological data on cocaine abuse suggest that, for unknown reasons, only a minority of abusers develop CIMDL. Specifically, considering the supposed rarity of CIMDL, it is possible that genetically determined predisposing factors could play a pivotal role in its genesis. 

This study is the first to investigate gene expression in CIMDL and found out that exposure of nasal epithelium to cocaine induces a diverse array of genes mainly related to apoptosis and autophagy/lysosomal activity, confirming that cocaine promotes apoptosis in exposed tissues [2,3,7].

In our CIMDL patient cohort 12 cocaine-induced genes bearing rare variants have been identified with a frequency greater than 80%, but none in all patients. As a consequence, a monogenic mechanism in CIMDL predisposition can be likely excluded. However, these observations do not rule out the possibility of genetic predisposition, which could still be present with involvement of multiple genes along common response pathways. 

The genes analyzed are involved in different cellular functions such as apoptosis and autophagy, tissue regeneration, cell proliferation, collagen integrity, and DNA damage response [19,20,21,22]. These findings support current knowledge about cocaine-induced tissue damage, which most probably results from oxidative stress and consequent apoptosis [7,23].

Nonetheless, in spite of already published evidence, widespread misconception remains about the pathogenesis of CIMDL, often attributed to mechanical irritation by cocaine crystals and hypoxic necrosis secondary to vasoconstriction, which are still mentioned in the literature as putative pathogenic mechanisms [24].

Among the 12 rare gene variants identified, 5 (OSGIN1, CACHD1, PPP1R15A, TBC1D2, PARP4) are related to autophagy and/or apoptosis. The latter is known to be induced by cocaine through generation of reactive oxygen species (ROS) that interact with DNA, mitochondrial membranes, and endoplasmic reticulum (ER), [6,23] and our findings confirm the importance of these processes in the pathogenesis of CIMDL [7,23]. Among the described genes, OSGIN1 and PPP1R15A appear to be the most closely associated with CIMDL, due to their direct implication with apoptosis. On the other hand, CACHD1, TBC1D2, and PARP4 could still be implicated, but their involvement in cell death and oxidative stress response appears less direct [25,26,27].

Other rare gene variants that were identified are not related to the response to oxidative stress, apoptosis, and autophagy, but rather to cell proliferation and tissue healing. Understandably, in the presence of ROS-related damage, not only pro-apoptotic or pro-autophagic molecular mechanisms play a role in the elimination of damaged structures and cells, but also regeneration is stimulated in response to injury and apoptosis [28] and could play an important role in the development of the CIMDL phenotype. 

The genes identified, namely AHNAK, C1orf116, FBN1, IQGAP1, PDLIM5, ZNF469, and PVR, are less decisively linked with CIMDL due to the absence of direct association with apoptosis or cell death. Additionally, it is difficult to hypothesize a clear mechanism of involvement in CIMDL pathogenesis for genes whose functions are poorly characterized, such as AHNAK [29] and C1orf116 [19].

The histological features of apoptosis are peculiar characteristics of CIMDL and represent one of the main tools useful to make a correct differential diagnosis with GPA, autoimmune pathology with similar clinical presentation compared to cocaine induced nasal lesions. In fact, both these conditions present histological features like mixed inflammatory infiltrates, microabscesses in vascular walls, perivenulitis, vascular microthrombotic changes, leukocytoclastic, vasculitis, and fibrinoid necrosis, [30,31] but only CIMDL presents massive apoptosis. In addition, the absence of extravascular changes (e.g., stromal granulomas with giant cells, microabscesses, and deeply located necrosis) peculiar to GPA, gives an additional clue for a correct differential diagnosis. Unfortunately, these histological differences are not always present and, in addition, CIMDL and GPA can share serological positivity for c-ANCA or p-ANCA [32,33]. These similarities are an additional challenge that can lead to incorrect diagnosis. However, recent studies have demonstrated that ANCA specificity for human neutrophil elastase (HNE), is peculiar of CIMDL and can be used, in addition to the histological feature of apoptosis, as a diagnostic tool [3]. At present, it is still unclear whether ANCA are active participants in the pathogenesis of CIMDL or, more probably, an epiphenomenon [2,3]. While it is theoretically possible that inflammation and immunity play a role in the development of CIMDL, it is unlikely to be the sole pathogenic mechanism, considering the experimental evidence gathered in the present study. 

This study presents some limitations. Firstly, while CIMDL patients, due to the progressive nature of their condition, [2] are likely to seek medical attention at some point, it is more difficult to study cocaine abusers without this condition. Indeed, repeating the same experiments on abusers without CIMDL might prove useful to identify relevant gene variants. Secondly, the rare variants related to addiction could co-segregate with those associated with CIMDL. Thirdly, it must be considered for all the selected genes that the increased frequency of rare variants in our population could be related to unknown selection bias. However, considering the known pathogenic mechanisms involved in CIMDL, it is reasonably possible to differentiate between variants related to each of the conditions. Fourthly, additional concern could rise if considered the “real” street dose of cocaine which is generally unknown due to the multiple times cut in the selling pipeline. However, our experiment deals with different concentration of cocaine, reducing, as much as possible, the influencing variability of cocaine dose. Fifthly, the gene expression of the nasal epithelium is limited to the acute effects of cocaine on the mucosa, while gene expression and immunological response resulting from chronic exposition to cocaine have not been investigated; indeed, the chronic effects of cocaine on nasal mucosa could be the subject of further studies. 

## 5. Conclusions

Our study demonstrated induced expression of a diverse array of pro-apoptotic genes in nasal epithelium exposed to cocaine. When considering the cohort of CIMDL patients, rare variants of 12 genes related to apoptosis, autophagy, tissue regeneration, cell proliferation, collagen integrity, and DNA damage response were expressed. Our findings, along with evidence from other studies, support the notion that oxidative stress is a crucial driver of damage affecting nasal mucosa exposed to cocaine crystals and, consequently, the most likely pathogenic mechanism of CIMDL. 

However, our experiments could not determine the relative importance of each expressed gene or molecular pathway in CIMDL and were limited by lack of data on disease prevalence, small sample size and absence of a control group of cocaine abusers without CIMDL. Determination of CIMDL prevalence and future experimental models with gene silencing could be helpful to delineate the molecular pathways relevant to CIMDL and elucidate its pathogenesis and the possible role of genetic predisposition.

## Figures and Tables

**Table 1:** Age, gender, and involved anatomical structures of the CIMDL patients included in the study.

Case Number	Gender	Age (Years)	Involved Anatomical Structures
1	Female	41	Nasal septum, middle, and inferior turbinates
2	Female	25	Nasal septum, middle, and inferior turbinates, lateral nasal wall, hard palate.
3	Female	38	Nasal septum, inferior turbinates
4	Male	45	Nasal septum, middle, and inferior turbinates, lateral nasal wall, soft palate.
5	Female	40	Nasal septum, inferior turbinates
6	Male	33	Nasal septum, middle, and inferior turbinates
7	Male	40	Nasal septum, middle, and inferior turbinates
8	Male	66	Nasal septum, middle, and inferior turbinates, lateral nasal wall, hard palate.
9	Male	39	Nasal septum, middle, and inferior turbinates
10	Female	32	Nasal septum, inferior turbinates
11	Female	31	Nasal septum, middle, and inferior turbinates, lateral nasal wall.

**Table 2:** Characteristics and processes of the twelve genes related to CIMDL. Case HET: Number of individuals carrying at least one heterozygous qualifying variant in the gene; Case HOM: Number of individuals carrying at least one homozygous qualifying variant in the gene; Case AC: Total Allele Count of qualifying variants in the gene; Control HET: Approximate number of individuals carrying heterozygous qualifying variants in the gene; Control HOM: Number of individuals carrying homozygous qualifying variants in the gene; Control AC: Total AC for the gene; *p*-value: *p*-value under the dominant model; Adjusted *p*-value: *p*-value corrected with Benjamini-Hochberg procedure; Processes: main function associated to gene.

Gene SYMBOL	Case HET	Case HOM	Case AC	Control HET	Control HOM	Control AC	*p*-Value	Adjusted *p*-Value	Processes
*AHNAK*	12	12	154	0	0	0	2.20 × 10^−49^	2.02 × 10^−45^	Cell adhesion
*C1orf116*	12	11	140	154	0	154	1.34 × 10^−31^	1.14 × 10^−27^	Wound healing
*CACHD1*	12	12	140	97	0	97	6.89 × 10^−34^	5.94 × 10^−30^	Apoptosis
*FBN1*	12	12	141	226	1	228	1.20 × 10^−29^	1.02 × 10^−25^	ECM formation, Cell adhesion
*IQGAP2*	12	12	281	135	1	137	3.21 × 10^−32^	2.75 × 10^−28^	Cytoskeleton
*PARP4*	12	11	374	1291	4	1299	1.08 × 10^−20^	8.78 × 10^−17^	Cell growth
*PDLIM5*	12	12	143	1267	10	1287	9.17 × 10^−21^	7.43 × 10^−17^	Cytoskeleton
*PVR*	12	12	138	0	0	0	2.20 × 10^−49^	2.02 × 10^−45^	Cell adhesion
*ZNF469*	12	12	443	321	0	321	6.98 × 10^−28^	5.90 × 10^−24^	Collagen formation
*OSGIN1*	11	4	171	848	9	866	7.88 × 10^−23^	6.46 × 10^−19^	Oxidative Stress, Apoptosis.
*PPP1R15A*	10	2	97	854	5	864	8.11 × 10^−23^	6.64 × 10^−19^	Apoptosis, Oxidative stress, wound healing.
*TBC1D2*	10	1	58	1435	10	1455	1.94 × 10^−17^	1.54 × 10^−13^	Autophagy

## Data Availability

Data are available from the authors upon reasonable request.
